# Intra-Urban Human Mobility and Activity Transition: Evidence from Social Media Check-In Data

**DOI:** 10.1371/journal.pone.0097010

**Published:** 2014-05-13

**Authors:** Lun Wu, Ye Zhi, Zhengwei Sui, Yu Liu

**Affiliations:** 1 Institute of Remote Sensing and Geographical Information Systems, Peking University, Beijing, China; 2 China Center for Resources Satellite Data and Application, Beijing, China; 3 Shenzhen Key Laboratory of Urban Planning and Decision Making, Harbin Institute of Technology Shenzhen Graduate School, Shenzhen, China; Inserm & Universite Pierre et Marie Curie, France

## Abstract

Most existing human mobility literature focuses on exterior characteristics of movements but neglects activities, the driving force that underlies human movements. In this research, we combine activity-based analysis with a movement-based approach to model the intra-urban human mobility observed from about 15 million check-in records during a yearlong period in Shanghai, China. The proposed model is activity-based and includes two parts: the transition of travel demands during a specific time period and the movement between locations. For the first part, we find the transition probability between activities varies over time, and then we construct a temporal transition probability matrix to represent the transition probability of travel demands during a time interval. For the second part, we suggest that the travel demands can be divided into two classes, locationally mandatory activity (LMA) and locationally stochastic activity (LSA), according to whether the demand is associated with fixed location or not. By judging the combination of predecessor activity type and successor activity type we determine three trip patterns, each associated with a different decay parameter. To validate the model, we adopt the mechanism of an agent-based model and compare the simulated results with the observed pattern from the displacement distance distribution, the spatio-temporal distribution of activities, and the temporal distribution of travel demand transitions. The results show that the simulated patterns fit the observed data well, indicating that these findings open new directions for combining activity-based analysis with a movement-based approach using social media check-in data.

## Introduction

The widespread use of location-aware devices, including smart phones and GPS (Global Positioning System) enabled cars, has provided powerful tools for collecting large volumes of time-resolved locations of individuals [1]. By exploring and analyzing the characteristics of huge amount of individual location data, intra-urban human mobility could be potentially depicted. At present, human mobility has received enhanced understanding from a wide range of fields, such as urban planning [2], [3], traffic forecasting [4], epidemiological models of disease spread [5], [6] and location-based recommender systems [7], [8].

Previous studies have concluded that intra-urban human mobility shows a high degree of temporal and spatial regularity. Far from being random, intra-urban mobility can be predicted by a number of factors [9], [10]. Furthermore, a number of analytical models have been proposed to explain and model the intra-urban human mobility patterns, including the gravity model [11], the generalized potential model [12], the intervening opportunities model [13], the rank-based movement model [14] and the radiation model [15]. In practice, these models have been operated from multiple perspectives such as geographical heterogeneity and distance decay [1], population density [15], geographical and social distances [16], urban morphology [3] and the spatial distribution of venues [14]. Such analyses can be summarized as movement-based approaches, which do not take into account the individual's travel demand. Because intra-urban mobility has not yet been closely inspected from an activity-based perspective, the diversity of travel demands that spur movement have been largely neglected [17]. In contrast, the activity-based approach treats travel demand as the driving force for human mobility, thus differentiating individuals from random walkers in exploring physical space [15], [18]. Moreover, since the sequence of activities determines the mobility patterns [17], this approach has brought about new perspectives on human movement in urban areas and has been widely used in transportation planning, i.e., to assess the impact of altered bus schedules [19] or to analyze the scaling laws for the movement of people [20]. However, due to the logistical restraints of recording activity information, much research on activity-based analysis currently is conducted through travel diary datasets collected by census and questionnaires on a small scale, resulting in both tremendous time and financial cost [21]. In order to capture the activities within an urban area, some research has utilized land use data information [4], [22], assuming that every basic land parcel keeps the same service function all the time. Of course, this assumption does not always conform to reality. One unit may satisfy various travel demands at the same time. Moreover, the major function of one cell may vary over time. For instance, one commercial cell could include restaurants, shopping malls and office buildings. Most consumers are likely to arrive at this zone for work in the morning and for shopping in the evening. Additionally, the activity-based analysis seldom considers the distance decay effect when exploring and evaluating the intra-urban human mobility patterns [23]. Although Hammadou et al. (2003) have measured the relationship between the distance decay and the activity-chain, they do not establish a reasonable model to explain the observed pattern [24]. Thus, a wide gap exists between activity-based analysis and movement-based approach.

Fortunately, since social media, such as Foursquare, Facebook, and Twitter, have been widely used, hundreds of millions of users have an ability to share their location and activity information by check-in data [25]. Different from cell phone data and car trajectories data derived from GPS trackers, check-in data has two unique features. First, check-in records not only contain the location but also include information about the user's motivation (what he/she is doing at the location). Second, the temporal check-in sequence of a specific person can be viewed as his/her trajectory. Although both the existence of fake check-ins, which occurs when users are not actually at or near the venues where they have checked in, and the limitation of age group (http://www.factbrowser.com/tags/foursquare/) would confine the scope of check-ins research on human mobility, check-in data has the ability to uncover human mobility according to some mechanisms [8]. Focus on check-in behaviors, a number of recent studies have been conducted. Scellato et al. analyzed the socio-spatial properties of individuals using check-in records [26]. Gao et al. integrated social-historical ties to model users' check-in behavior [27]. Pelechrinis et al. and Preo et al. studied the patterns across activities transition for check-ins [28], [29]. However, existing research does not pay much attention to temporal activity characteristics and their underlying geographical constraints. Our paper emphasizes the fact that check-in data has the capacity to bridge the gap between activity-based analysis and movement-based approaches in modeling intra-urban human mobility.

In this paper, more than 15 million social media check-in users are investigated during one year in Shanghai. We find that the successor activity of an individual varies over time and is affected by the predecessor activity purpose and time. Additionally, the activity can be divided into two classes: locationally mandatory activity (LMA) and locationally stochastic activity (LSA), according to whether the demand is associated with a fixed location or not. As a result, there are three kinds of trips depending on different combination of activity types. After analyzing distance distribution of the three trip patterns, we discover these patterns differ in their distance decay exponents. To interpret the observed patterns, we construct an activity-based model that integrates both activity-based and movement-based approaches. Adopting the mechanism of agent-based modeling, the result shows that the simulated patterns fit the observed data well.

## Materials

### 1. Dataset

**Figure 1:**
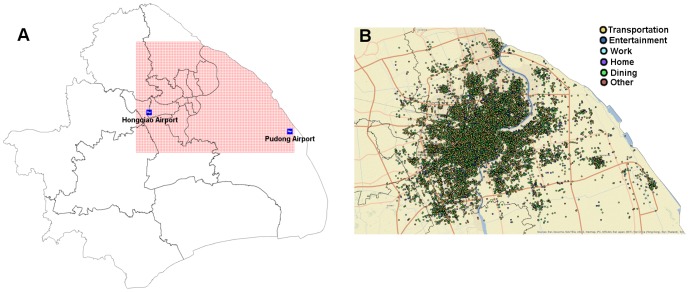
Spatial distribution of check-ins and the study area. (a) The study area in Shanghai. The red lattices represent the study area, and covers two airports, Pudong airport and Hongqiao airport. (b) Spatial distribution of check-ins by activities in the study. One check-in record is geo-referenced as one point according to its location. Different colors of the points denote different activities.

Analysis on the intra-urban movement is extracted from 15,213,360 social media check-in records of 257,278 users across 97,324 venues collected during the yearlong period from September 2011 to September 2012 in Shanghai. The data used in this study can be shared with other researchers upon request. These records are also part of the check-in data set that has been previously applied in an analysis of inter-urban trip and spatial interactions [30]. Because each check-in is not only associated with a specific geo-tagged venue (e.g. restaurants, shopping malls, airport terminals and schools) but also correlated with a precise geographical coordinate attribute including latitude and longitude, the user's demand of movement can be identified. By considering the heterogeneous distribution of check-ins, we choose the central part (50×35 km^2^) of the city for the study (Figure 1a) and visualize the spatial distribution according to different activity types (Figure 1b).

### 2. Filtering Check-in Records

**Figure 2:**
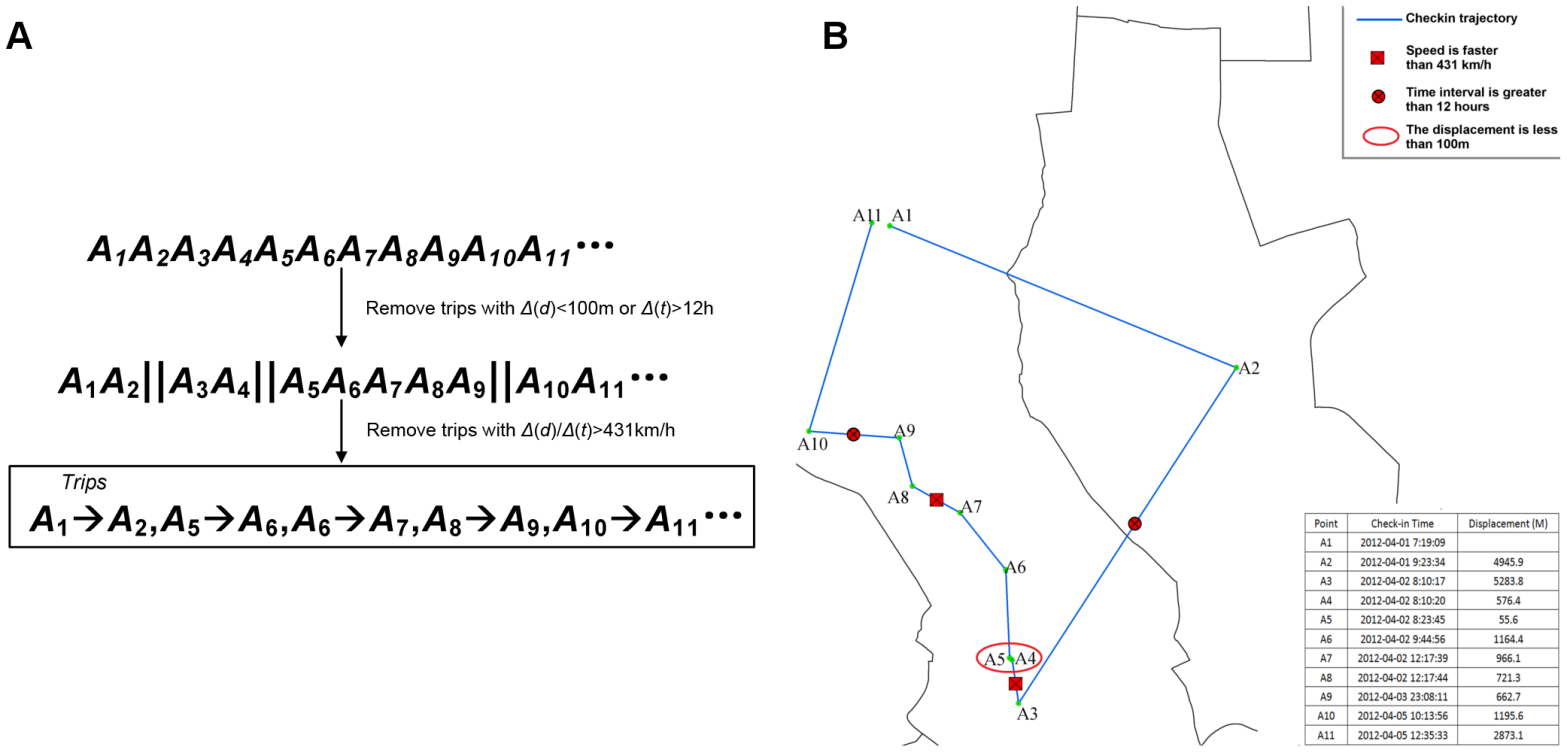
Criteria for extracting trips. (a) Two steps for extracting trips from one individual check-in trajectory. A_1_A_2_A_3_A_4_A_5_A_6_… is one individual trajectory sequence. (b) The demonstration of applying the criterions into the anonymous individuals' trajectories. The blue line is the original check-in trajectory. When segmenting this trajectory to trips, we filter the successive check-in pairs that the speed is faster than 431 km/h, such as A_3_->A_4_ and A_7_->A_8_; or time interval is greater than 12 hours, such as A_2_->A_3_ and A_9_->A_10_; or the displacement is less than 100 m, such as A_4_->A_5_.

Although most social media services provide some mechanism to prevent the emergence of fake check-ins, invalid check-ins and trips still exist. For some reason, a person staying home may post a check-in record indicating that he (or she) is at a restaurant. These instances hamper the usefulness of data for exploring intra-urban human mobility patterns and must be eliminated because of the discontinuous characteristic of their check-in sequence. We proposed five criteria to filter out the fake check-ins and trips: (i) the location of check-in is not in the study area; (ii) the distance between the location of declared check-in venue and the location of user's cellphone GPS coordinates is greater than 500 m; (iii) the user who has only one check-in. After extracting each individual spatio-temporal trajectory (consecutive check-ins), we segment the trajectories to trips datasets and remove the anomalous trips according to the following criteria (Figure 2a): (iv) the length of displacement is less than 100 m or the time interval is greater than 12 hours (Intuitively, if the time interval is greater than 12 hours, these two activities are regarded as a low correlation and should be segmented into different trips.); (v) the rate of speed is faster than 431 kilometers-per-hour (or faster than a maglev train). As displayed in Figure 2b, the original individual's check-in trajectory is comprised of eleven check-in points. According to the above criteria, only five trips are finally obtained.

### 3. Determining Lattice Size

**Figure 3:**
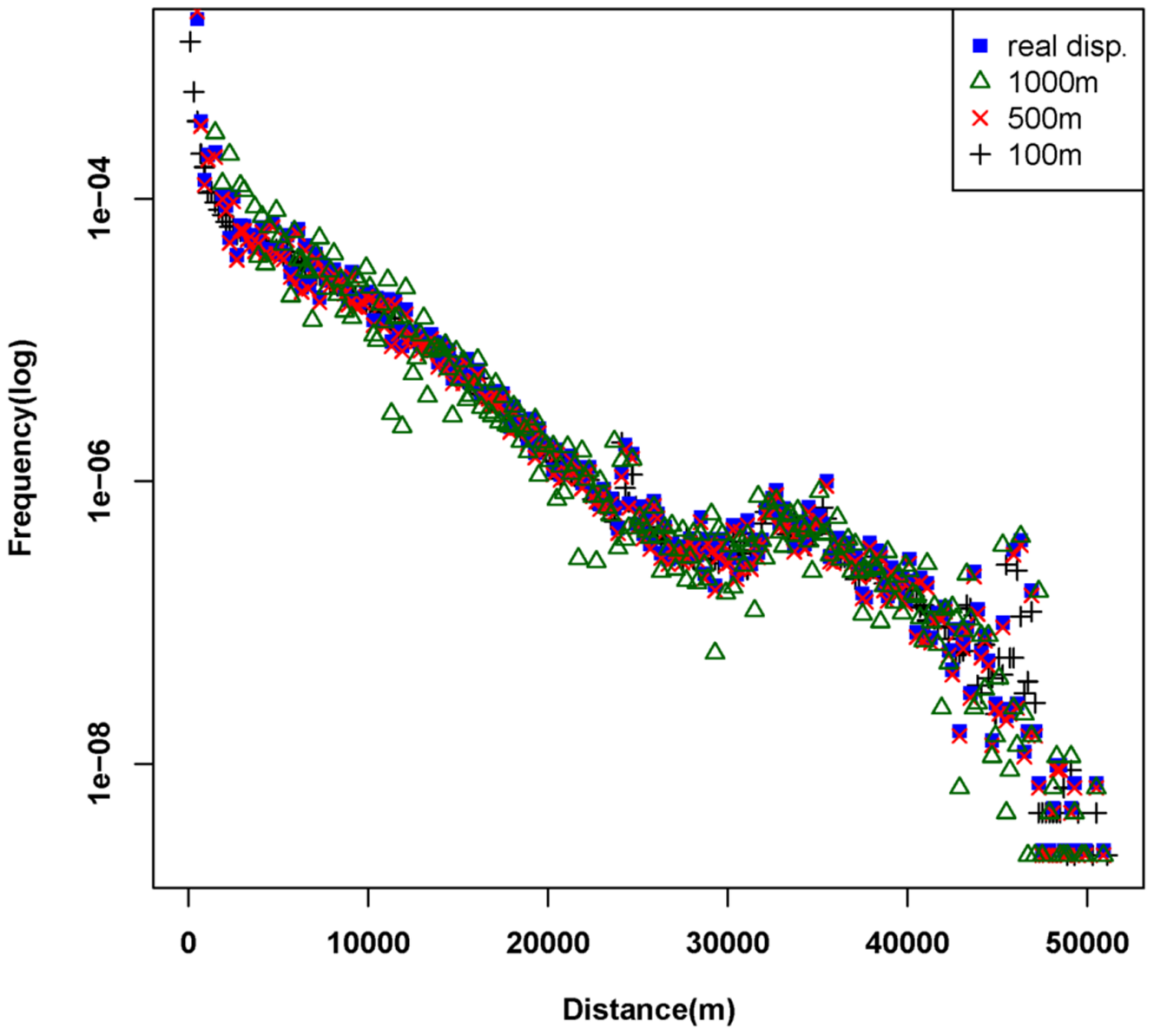
Distribution comparison between distances approximated in different lattice sizes and actual distances. The 1000's.

When analyzing the check-in data, the urban area is divided into square lattices, and a trip length can be approximated by the distance between centers of cells that the predecessor and successor points are placed in. The merit of this approach is that we can construct a continuous representation for human movements so that the interpretation model can be built. Obviously, the deviation of the trips' displacement will become larger and larger with the increasing size of the lattice. However, if the size is too small, it is inappropriate due to relatively increasing computing costs and also because the patterns among different regions are random and unclear [5]. As shown in Figure 3, if the size of lattice is greater than 500 m, the deviation is obvious comparing to the real distribution of trip displacements. So the lattice cell size is fixed as 500 m in this paper.

### 4. Categorizing Travel Demands

The check-in data have the advantage over other GPS-enabled data (such as taxi trajectory data or mobile call records) in indicating the purpose of individual travel with the help of demand-tags. However, some demand-tags signify a similar purpose: for example, dining can be expressed as western food, Chinese food, snacks, fast food and so on. Therefore, the categorization of travel demand is indispensable for the emergence of regular mobility patterns.

Much research on the categorization of travel demand (or the type of venue) for intra-urban human mobility has been conducted. Bagrow and Lin classified the travel destinations as residential subdivision, government office, hospital, school, park, shopping place (including shopping malls, super markets, etc.), hotel, restaurant, and factory [6]. Similarly, the travel demands may be regarded as one of the following types: residential areas, workplaces, commercial areas, recreational areas, educational places and transportation [2]. Ben-Akiva et al. simplified the categorization to be residences, workplaces and others [31]. Moreover, Ye et al. analyzed the temporal-sematic interaction for each travel demand [32]. In this research, considering the temporal characteristic of travel demand [6], we group the travel purpose into six categories: home (H), transportation (Tr), work (W), dining (D), entertainment (E) and other (O).

## Methods

### 1. Model Framework

Let M = {m_1_, m_2_, …} denote the domain of travel demands, and *T* = {*t*_1_, *t*_2_, …} denote the collection of time intervals. As the study area has been assumed to be divided into squares (the locations), each square could be marked with a certain number ranging from left to right and from bottom to top, represented as *L.*

Hence, one activity A is defined as a triple (*m*, *l*, *t*), where 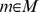, 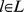 and 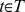, so that an individual trajectory could be represented as a sequence of activities {*A*_1_, *A*_2_, …}. In general, the trajectory is also regarded as the collection of trips [1], [30]. Similarly, in our model, we also segment the trajectories into trips datasets *R* = {*R*_1_, *R*_2_, …}.

One trip *R_k_* is defined as a vector including two activities <*A_kp_*(*m_kp_*, *l_kp_*, *t_kp_*), *A_ks_*(*m_ks_*, *l_ks_*, *t_ks_*)>, where *A_kp_* is the predecessor activity, and *A_ks_* is the successor activity, 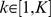, 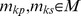, 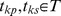, 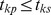 and 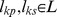; *K* is the number of trips. If not taking into account location, a trip *R_k_* could be viewed as a transition between two travel demands with temporal information, for which use the term time-dependent travel demand (TTD). A typical example of TTD is “shopping in the afternoon”. The transition between two TTDs is defined as 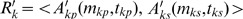.

In order to interpret the observed movement patterns, a model that integrates both the activity-based and movement-based approaches is proposed. We assume that the probability of the transition between TTDs (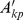 and 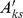) is location independent, thus the transition probability between two activities *A_kp_* and *A_ks_*, denoted by *P*(*Tp_A_*), could be decoupled into two parts, the transition probability *Tp_M_* between TTDs during the specific time period and the transition probability *Tp_L_* between locations. When the successor travel demand *m_ks_* and successor time *t_ks_* have been identified (or 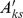 has been identified), the individuals then will determine the successor location *l_ks_*. Hence, the probability of the transition between two activities *A_kp_* and *A_ks_* is denoted by:
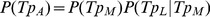
(1)or
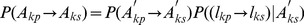
(2)First we focus the 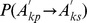. Transitions between travel demands have been previously studied in human movement [29], [33] and assumed that transition probability from one travel demand to another was not influenced by time. However, this proposition does not always match with reality. Both the demands for breakfast and supper can be regarded as dining. However, a person is likely to go to the workplace after breakfast but look for entertainment after supper. Hence, we have to take into account the time dimension and use the TTDs instead of time-independent travel demands. We defined the frequency of a TTD transition 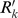 in the collection 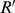 as the variable 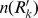 or 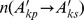, thus, the transition probability between two TTDs during a specific period is denoted as, 
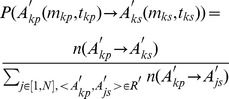
(3)indicating that the probability of occurrence for successor TTD *m_ks_* at successor time *t_ks_* is conditioned by *m_kp_* at time *t_kp_*.

In terms of the transition probability 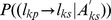 between locations, intuitively, activities could be divided into two classes, LMAs and LSAs, according to whether the travel demand associates with fixed location or not. For example, home and workplace are always attributed with the fixed location for an individual in his/her daily movements. On the contrary, dining and entertainment sites are always attributed with multiple alternatives for an individual to choose.

Thus the type of demand of an activity is defined as 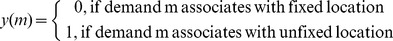
(4)Moreover, LMA and LSA trips would be affected by different factors when one person chooses his/her successor activities. For example, when one goes for lunch, he/she is likely to choose a closer restaurant from a number of candidates. However, when the person goes home, the destination is determinate, no matter how far it is. Hence, we assume that LMAs would consider the locational transition probability. On the contrary, we suggest that the transition probability of locations is not only affected by distance decay but also geographical heterogeneity for the LSAs. Previous research has indicated these effects for analyzing of human mobility patterns. Liu et al. introduced the population density data to represent geographical heterogeneity in mobility demand, and used this data set to simulate mobility patterns within Shanghai [1]. Similarly, Liang et al. utilized the distribution of origins and destinations instead of population distribution in another mobility simulation [5]. But both of them did not take into consideration the time dimension and the travel demands and only focused on predicting traffic flows from one grid to another at the collective level. Differently, we want to explore these effects at the individual level and factor the temporal spatial intensity distribution of each LSA into the overall model of geographical heterogeneity. When the successor temporal demand 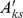 is known, the spatial distribution of locations, where travel demand *m_ks_* would be satisfied at time *t_ks_*, can be obtained. However, these candidate locations will differ in the intensity of travel demand and the distance from the user's current location. For example, given a travel demand such as shopping, a number of places, including supermarkets, shopping malls, and stores, are available. They are with different sizes and locations, both of which influence the individual's choice of next trip. For the sake of computation, the study area can be discretized in to square pixels. In each squares *l_ks_*, the capacity for satisfying successor TTD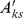 is denoted as 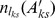, where 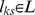. After this, the distance between the location of predecessor activity *l_kp_* and the location of candidate activity 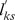 can be identified as 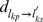
and the distance decay is represented by 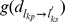. Thus, the transition probability of locations is represented as
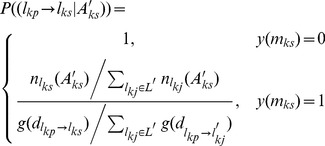
(5)where 
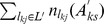
 represents the total number of 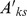 in *L*′ and 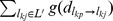 is the sum of distances from *l_kp_* to all locations in *L*′.

As a result, the probability of the transition between two activities *A_kp_* and *A_ks_* is finally denoted by
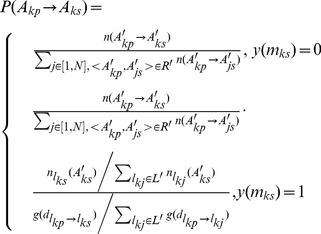
(6)


In sum, Equation 6 indicates that LMA trips only take into account the transition probability between TTDs while LSAs allow for not only the transition probability between TTDs, but also the capacities for satisfying the successor TTDs of all sites and the distance decay effect.

### 2. Simulation and Evaluation

In order to verify our model, it is suggested that agent-based modeling (or individual-based modeling) [34] be adopted to reproduce the observed human mobility patterns. Agent-based modeling has been widely applied to simulate transportation patterns [20], [35], emergency evacuation [36] and urban sprawl [37], because this approach can simulate the individual actions in time series and measure the outcome for the analysis of mobility patterns[38]. In the simulations, each individual is considered as one agent with an initial status, and that agent will determine the next activity according to Equation 6, when its current activity has been completed. Note that Equations 6 yields probabilities and we introduce the Monte Carlo method to deal with randomness. The output is a dataset including each agent's simulated activity trajectory. After segmenting the simulated activity trajectories into trips, we can compare the simulated mobility patterns with the observed ones.

To evaluate the similarity between the simulated data and the observed data, the Hellinger coefficient is adopted [39]. The probability density functions of two continuous distributions are supposed to be p(x) and q(x) within the same domain X. Then the Hellinger coefficient is given as follows:
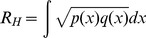
(7)For discrete distributions, the equation is denoted as:
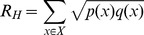
(8)


## Results

In this work, the number of demand categories is |*M*| = 6, and the number of time intervals is |*T*| = 24 since one hour intervals are adopted as the temporal unit for analysis. The study area has been divided into 500×500 m^2^ squares, and the total number of squares is |*L*| = 5836 after filtering out water areas. By removing noise check-ins, 2,230,366 trips are extracted from the entire dataset, meaning |*R*| = *K* = 2,230,366. With regard to LMAs and LSAs, the demands for Tr, H and W are regarded as the LMA, and the demands for D, E and O are considered as the LSA. Note that each individual in general has a fixed mode for transportation in daily life, and thus Tr is assumed to be a LMA.

### 1. Spatio-Temporal Distribution of Different Activities

**Figure 4:**
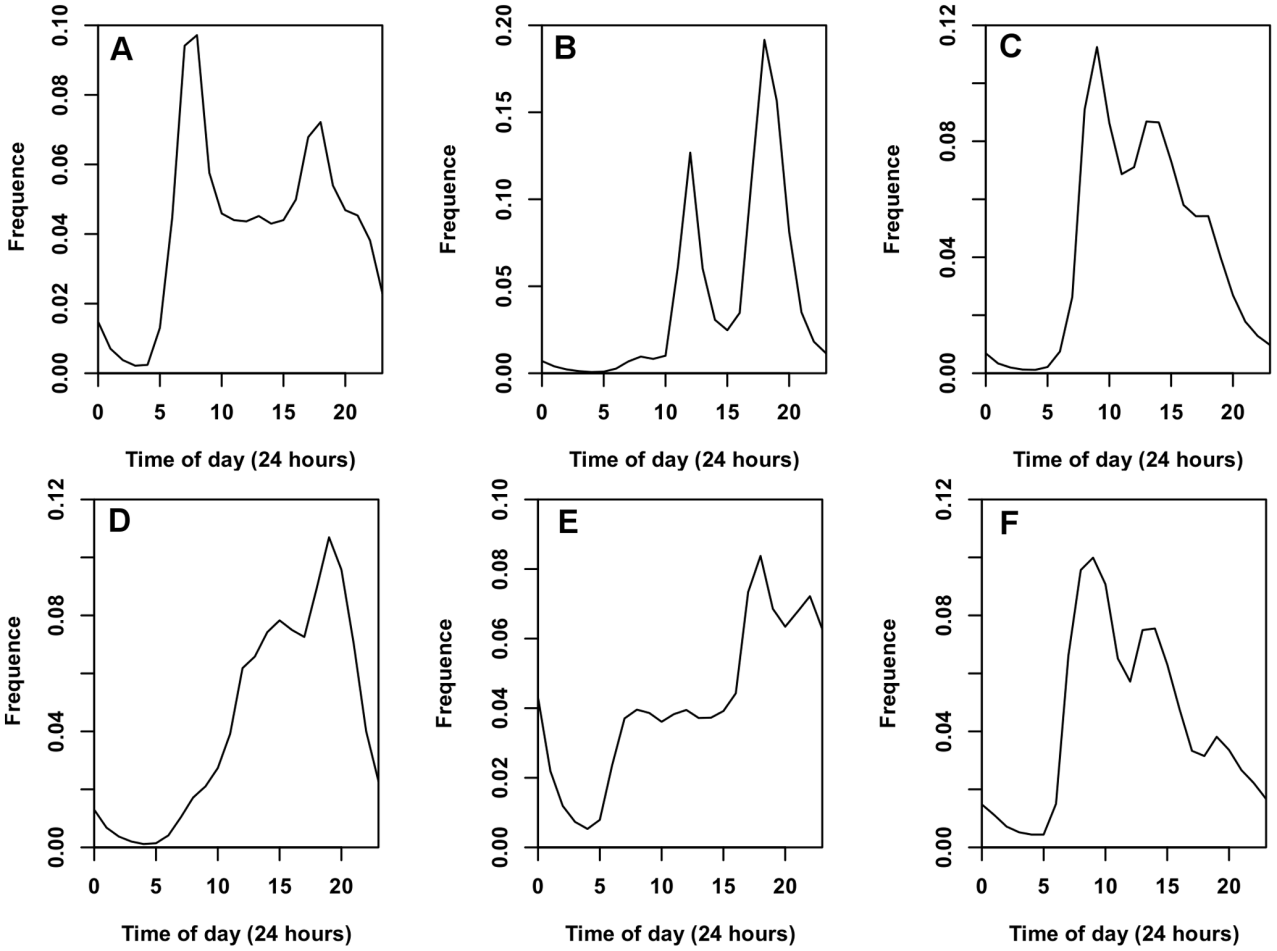
Diurnal temporal distribution of different activities. a) Transportation. b) Dining. c) Work. d) Entertainment. e) Home. f) Other. The frequency curves of Tr, D, and W each have two peaks that emerge during different periods throughout the day. The first peaks for both Tr and W appear in the period from 7 am to 9 am; at lunchtime, the D reaches its first peak. The W's second peak is earlier than the other two's. The trend lines for both E and H remain at a low level during the daytime and rise after 5 pm. The curve of O is almost same as the W's.

**Figure 5:**
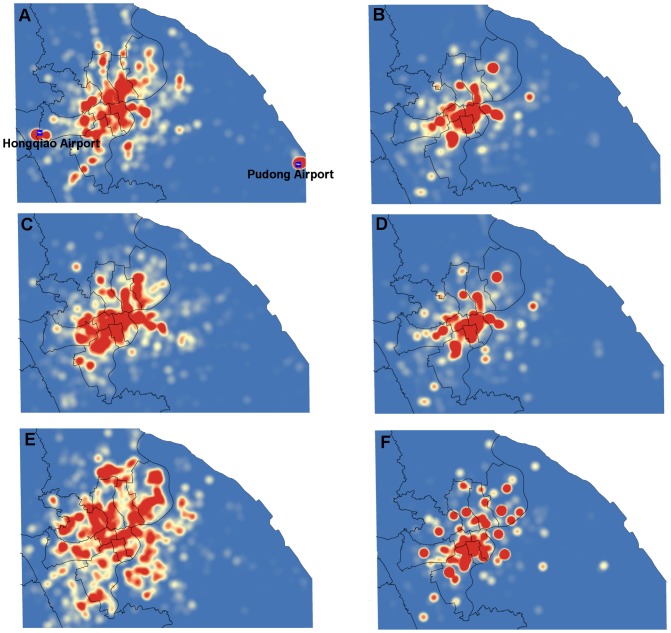
Spatial distributions of different activities. In order to make the spatial distribution more clear, the kernel density estimation (KDE) method is adopted. a) Transportation. b) Dining. c) Work. d) Entertainment. e) Home. f) Other. The demands for W, D, E and O are mainly accumulated in the central area, but the demand O is more discrete than the other three. Tr has two special hot spots, which are the Pudong airport and the Hongqiao airport.

The travel purposes are grouped into six categories, as shown in Figures 4 and 5, each travel purpose has unique temporal and spatial distribution characteristics, which are consistent with common knowledge. We observe that the Tr, D and W each have two peaks that emerge during different periods throughout the day. The first peaks for both Tr and W appear in the period from 7 am to 9 am; at lunchtime, the D reaches its first peak. The W's second peak is earlier than the other two's, suggesting that most of residents are likely to go back to the office after lunch. The trend lines for both E and H remain at a low level during the daytime and rise after 5 pm, showing that the majority of users will return home or participate in entertainment after work. In our method, the travel purpose for school, public library, and the attraction sites are merged into O, which looks the same as the W. From the perspective of spatial distribution, the demands for W, D, E and O are mainly accumulated in the central area. But we observe that the O is more discrete than the other three, which is probably because the places for O are generally scattered. Particularly, Tr has two special hot spots, which are the Pudong airport and the Hongqiao airport. In summary, these six categories are proved to be good qualitative and descriptive explanations for intra-urban human mobility demands.

### 2. Transition Probability Matrix between Travel Demands

**Figure 6:**
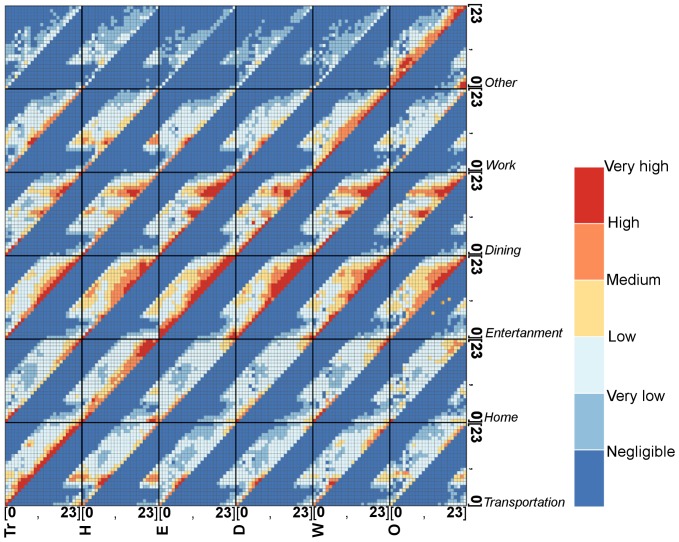
Temporal transition probability matrix of activities. The horizontal axis is the predecessor demand and time, 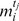
and the vertical axis is the successor demand and time, 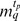. The transition probability is negligible if the successor time is twelve hours greater than the predecessor time. Obviously, the values for both the dining and entertainment demands during the 7 pm to 9pm from other demands are high. Especially, a high transition probability exists if the successor activity is entertainment at time from 7pm to 9pm on the condition that the predecessor activity is dining at time from 6pm to 7pm.

According to Equation 3, we construct a transition probability matrix *M_d_* between TTDs. The size of *M_d_* is *N*×*N* with *N* = |*M*|×|*T*|. The value of each cell in matrix *M_d_* is represented as
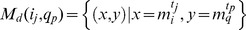
(9)The column unit of *M_d_* is the predecessor activity *m_i_* at time *t_j_* and can be denoted as 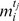, where 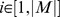 and 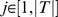. Similarity, the row unit of *M_d_* is the successor activity *m_q_* at successor time *t_p_* and is denoted as 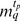, where 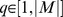 and 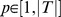. Thus, the cell of *M_d_* with index (*i_j_*,*q_p_*) records the frequency of occurrence for successor activity *m_q_* at successor time *t_p_* conditioned on predecessor activity *m_i_* at predecessor time *t_j._*, and the *M_d_* is visualized as shown in the Figure 6. The value of cell, for example, (E_19_, D_20_) equals to 0.08, indicating that the transition probability from the predecessor activity E in the 19th time interval (i.e. from 19:00 to 20:00) to the successor activity D in the 20th time interval is 0.08. Since the maximum time interval of a trip is set to twelve hours, the transition probability is negligible if the successor time is twelve hours greater than the predecessor time (the dark blue parts). From the vertical view (from the bottom to the top), the percentage of successor demands in the same time intervals can be obtained. For instance, the probabilities for treating entertainment and dining as the successor demands are relatively higher than other demands during the evening and at night. Likewise, from the horizontal view (from left to right), we can compare the percentage of their predecessor demands in the same time intervals. For example, although the transition probabilities for all the predecessor demands to the successor demand for dining are high during the evening and at night, the entertainment exposes much higher percentage than other demands.

### 3. Displacement Distributions of Different Trip Types

**Figure 7:**
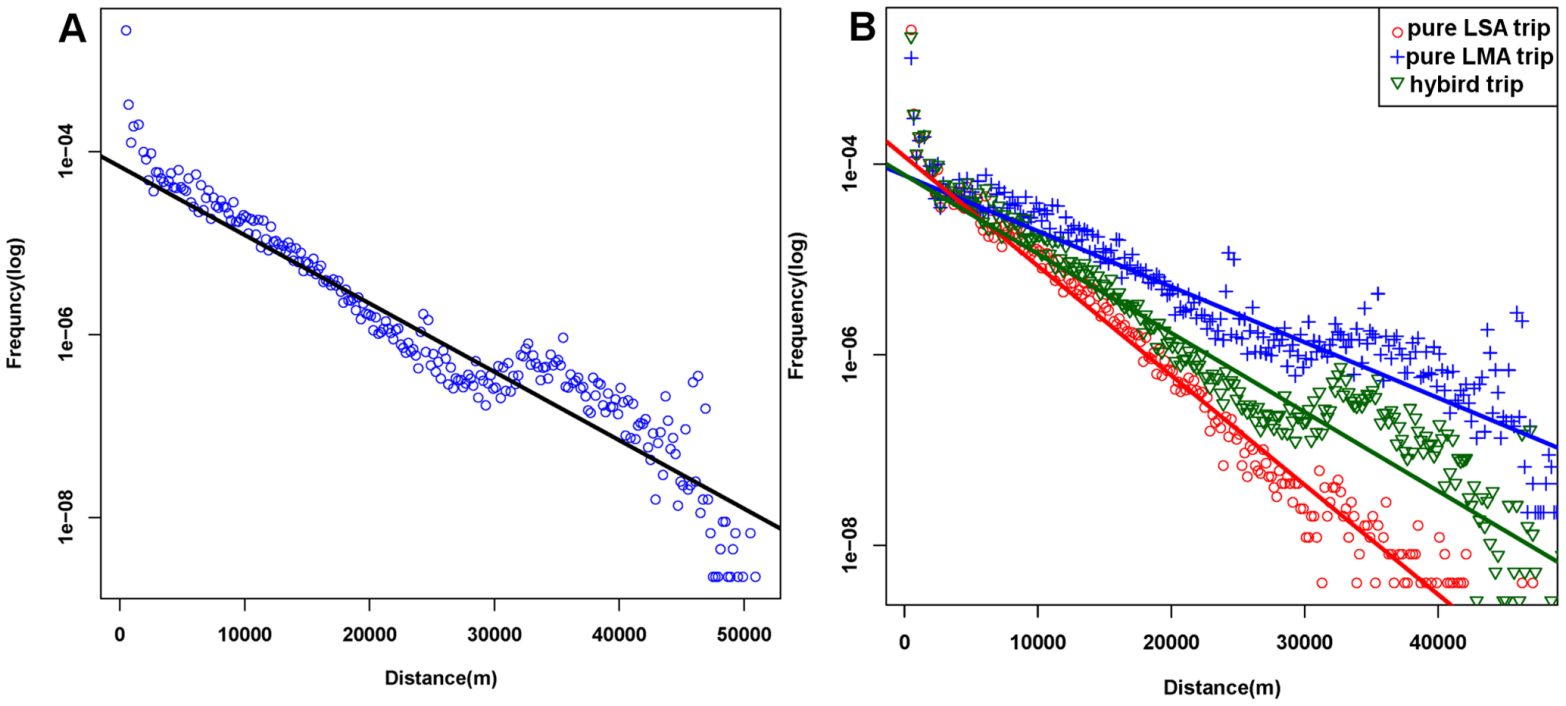
Distribution of trip distances. A) The distance distribution of all trips. B) The distance distribution of three trip patterns. The exponent of pure LMA trips is 0.134 km^−1^ (R^2^ = 0.713) whereas the pure LSA's is 0.264 km^−1^ (R^2^ = 0.9312). The exponent for hybrid pattern is 0.191 km^−1^ (R^2^ = 0.814).

To verify the hypothesis that LMA and LSA would be affected by different factors when one person chooses his/her successor activity, the displacement distribution *P*(Δ*d*) is investigated. *P*(Δ*d*) plays a basic statistical role in characterizing human mobility and is considered to be affected by not only the distance decay, but also other factors, such as geographical environments [1] and population heterogeneity [40]. We assume that the spatial distributions of both LSAs and LMAs are influenced by the same geographical and demographic factors at the macro scale. Hence, LSAs and LMAs will illustrate different characteristics when comparing their displacement distributions. Currently, two models are often used to fit *P*(Δ*d*): a power-law *P*(Δ*d*) ∼Δ*d* ^-β^ and an exponential law *P*(Δ*d*)∼exp(-*λ*Δ*d*). In terms of urban areas, recent research has demonstrated that the displacement distribution obeys exponential law rather than power-law according to mobile phone records [30], individual vehicle data [41] and taxi data [42]. Similarly, as Figure 7a shows, the distribution of individuals' movement in check-ins also follows roughly a straight line on a log-linear plot and reveals an exponential law with *λ* = 0.179 km^−1^ (R^2^ = 0.922).

However, a small peak exists between 30 km and 40 km, which corresponds with the result observed from taxi trajectories in a previous study of the Shanghai urban area. This phenomenon could be ascribed to the location of the Pudong International Airport [1]. Owing to more than 30 km away from the center of Shanghai, the airport makes residents travel long distances without other choices. With the respect to the distance decay, this peak also reflects that some activities are not affected by the distance decay to some extent. Therefore, it is necessary to divide the activity into two classes according to whether the demand associates with fixed location or not, thereby there are three trip patterns based on the types of the predecessor activity and the successor activity. If both the activities are classified as LMAs, the trip pattern is regarded as a pure LMA trip. Likewise, the trip pattern is considered to be pure LSA if both predecessor and successor activities can be classified as LSA. Last, if the two kinds of activities are different, the pattern can be deemed as a hybrid trip. As displayed in Figure 7b, the distribution of the pure LSA trip distance is more sharply decayed than the other two and have a very good fit for the exponential law with exponent *λ* = 0.264 km^−1^. Both the pure LMA and pure LSA patterns have hardly any peak, and more importantly, the plot reveals that the human mobility with different trip patterns will be affected by different distance decay effect.

Hence, the hypothesis that LMA activity would not be affected by the distance delay is proved to be correct for interpreting the intra-urban human mobility when one person chooses his/her next activity. Therefore, if the successor demand is locationally mandatory, the person will get in a specific location directly without the transition probability of locations' issue.

### 4. Simulation Results

After considering the computing cost, we initialize 120,000 virtual agents and randomly place each of them into a 500×500 m^2^ square designated as the agent's home, according to the population distribution of Shanghai, for which we use the LandScan 2008 High Resolution Global Population Data Set (http://www.ornl.gov/sci/landscan/). Then each of the agents' activity is assigned from the collection *M* at random. Finally, we set the beginning time to 6 o'clock and make each agent individually assess their respective situations and move according to the proposed model. For LSA, we adopt the frequency of check-in which can fit the successor TTD to represent the capacity of satisfying successor TTD in each square. During the simulation, given the user's current location, some trivial but close places may exist. Since we use a power-law distance decay function, *d* ^−*β*^ is rather high when *d* is small and thus overestimates the impacts of such places. Therefore, we adopt a threshold *μ* to filter squares that have lower frequencies of TTD than *μ* when an individual chooses the next stop. In this research, *μ* is set to 10 by trial and error. Additionally, we simplify the relationship between the distance decay versus the activity transition and utilize the same distance decay function *g*(*d*), since Liang et al. suggested that the power law functions are more in accord with the reality than the exponential functions in the simulation [5]. Likewise, Liu et al. pointed out that the observed displacement distribution of intra-urban trips can been well interpreted using a power law distance decay function [1]. Hence, we set *g*(*d*) to be *d* ^−β^ in the simulation, where β is the distance decay parameter. Different exponent values between 1.0 and 2.0 were tried, and about 2,100,000 trips were generated for each exponent. We found the observed pattern could be best fitted when β = 1.62. Finally, we segmented the agents' simulated activity trajectories into trips and compared them with the observed ones from displacement distribution, spatial and temporal distribution and TAD distribution.

**Figure 8:**
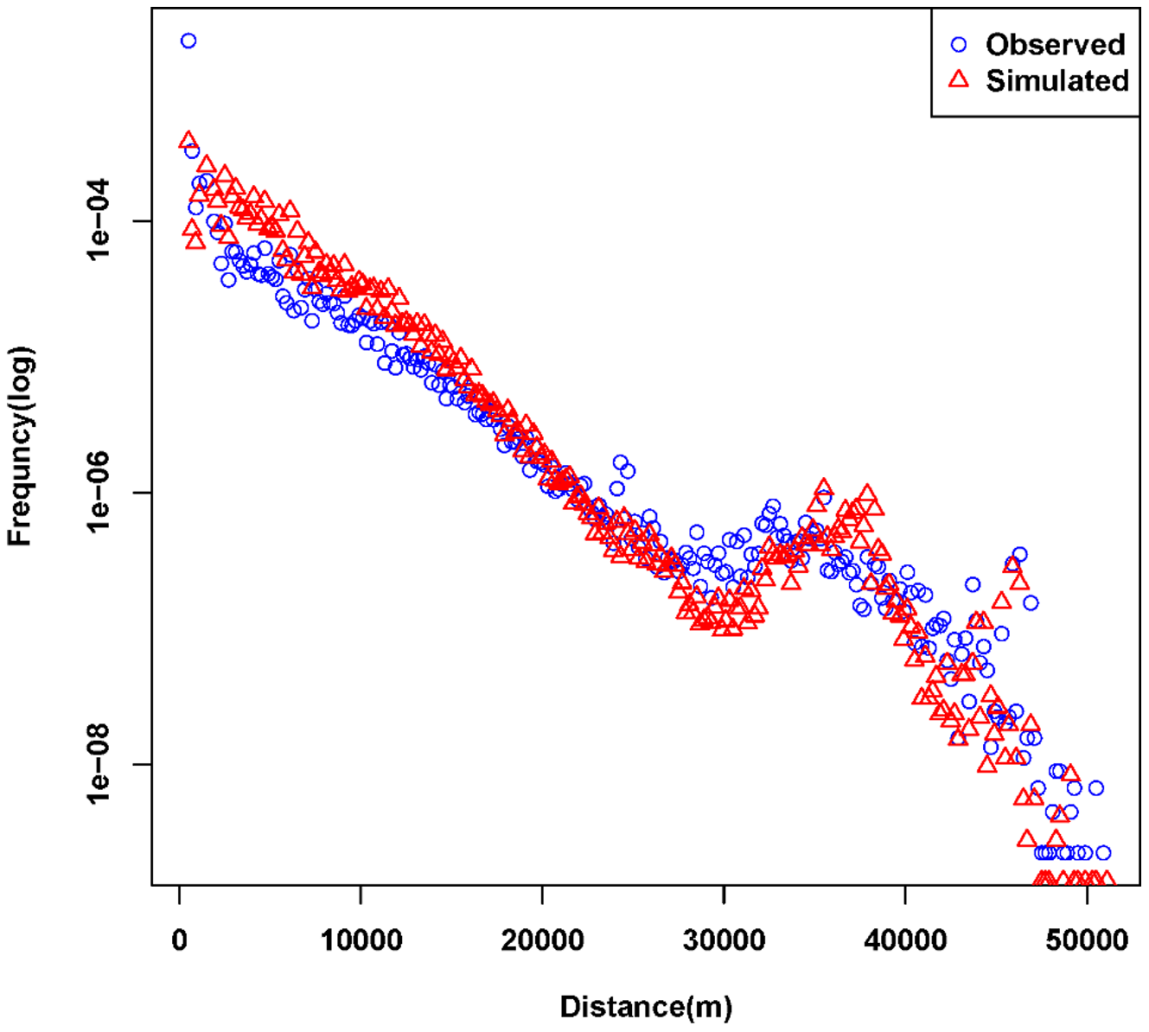
Comparison between distance distributions of observed and simulated trips. The Hellinger coefficients is 0.8829, and a peak also exists between 30

**Figure 9:**
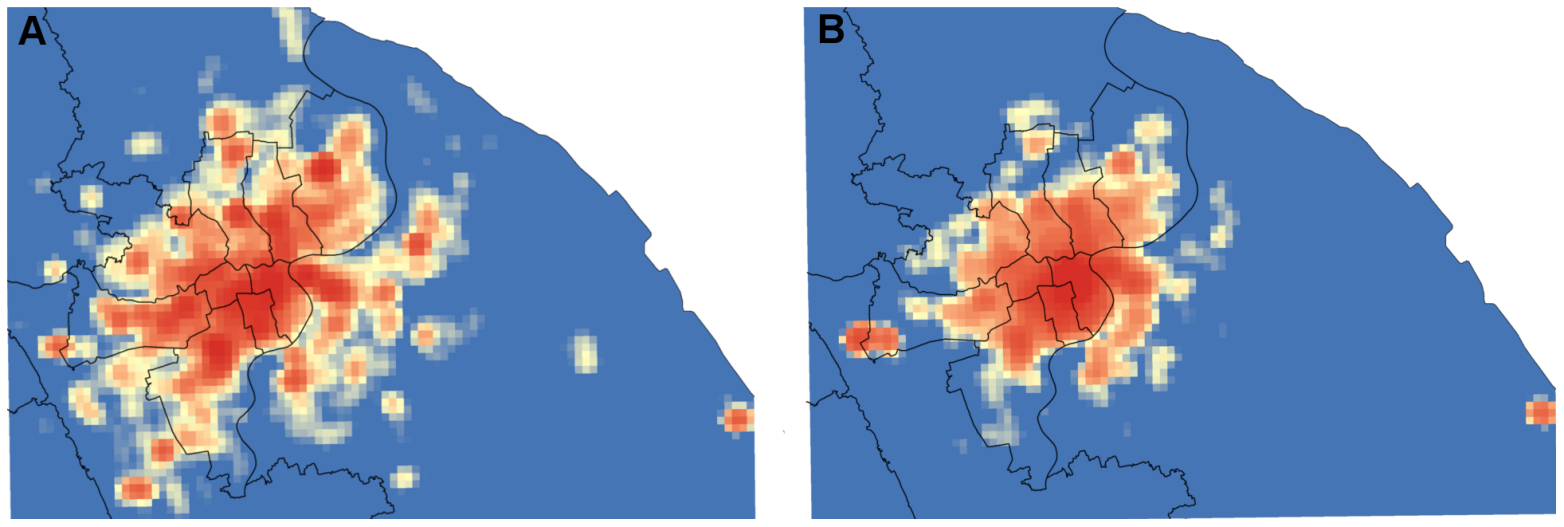
Comparison between spatial distributions of observed and simulated trips. The KDE method is adopted, and the output cell size is 250,000 square meters. a) The observed successor activities. b) The simulated successor activities. The vast majority part of the observed data can be illustrated by the simulated one, and the Hellinger coefficient is 0.8430.

**Figure 10:**
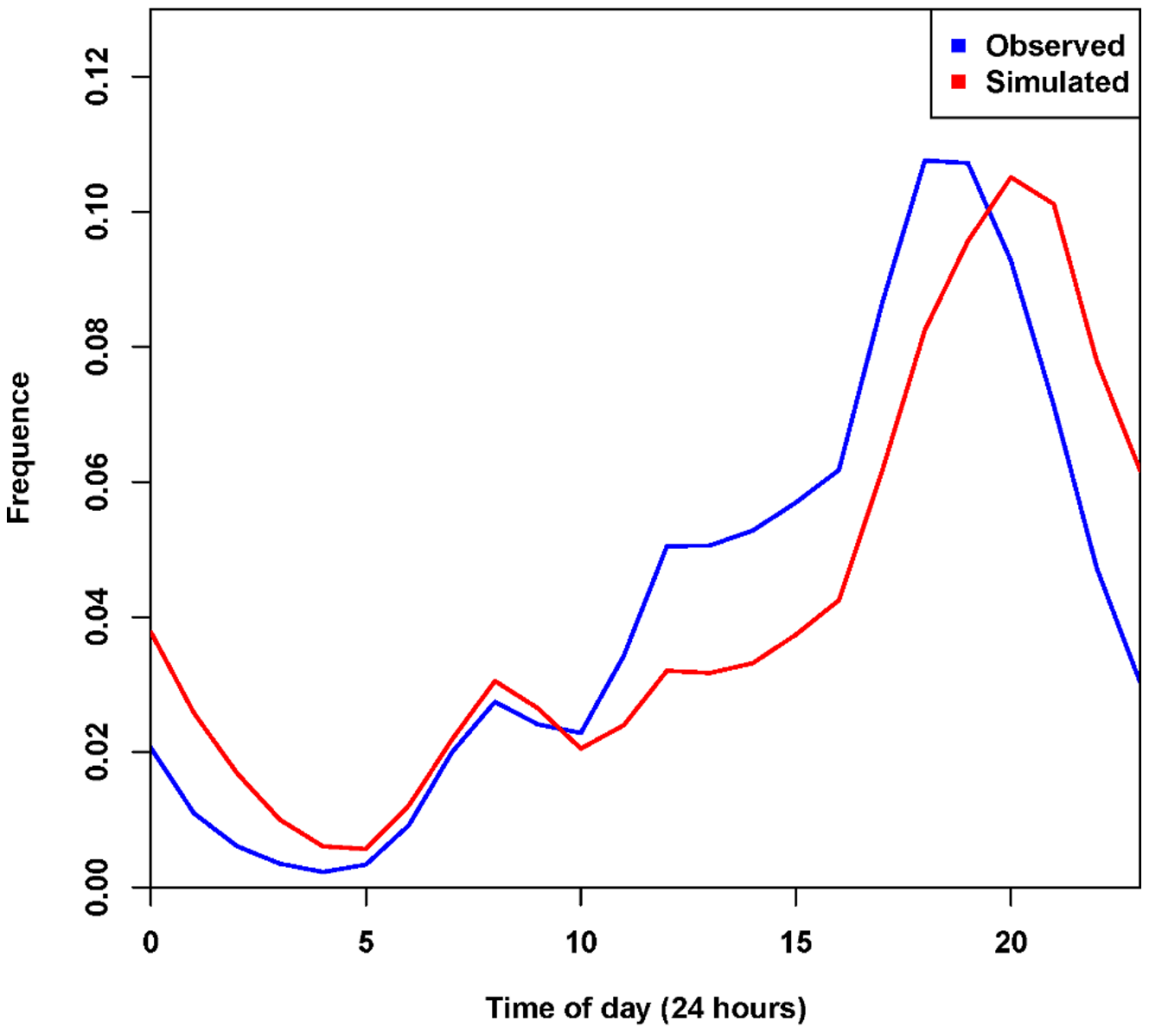
Comparison between temporal distributions of observed and simulated trips. The Hellinger coefficient is 0.9803. In evening time, we can find a one-hour lag exists between two peaks. The lag should be attributed to the one-hour temporal resolution in simulations.

**Figure 11:**
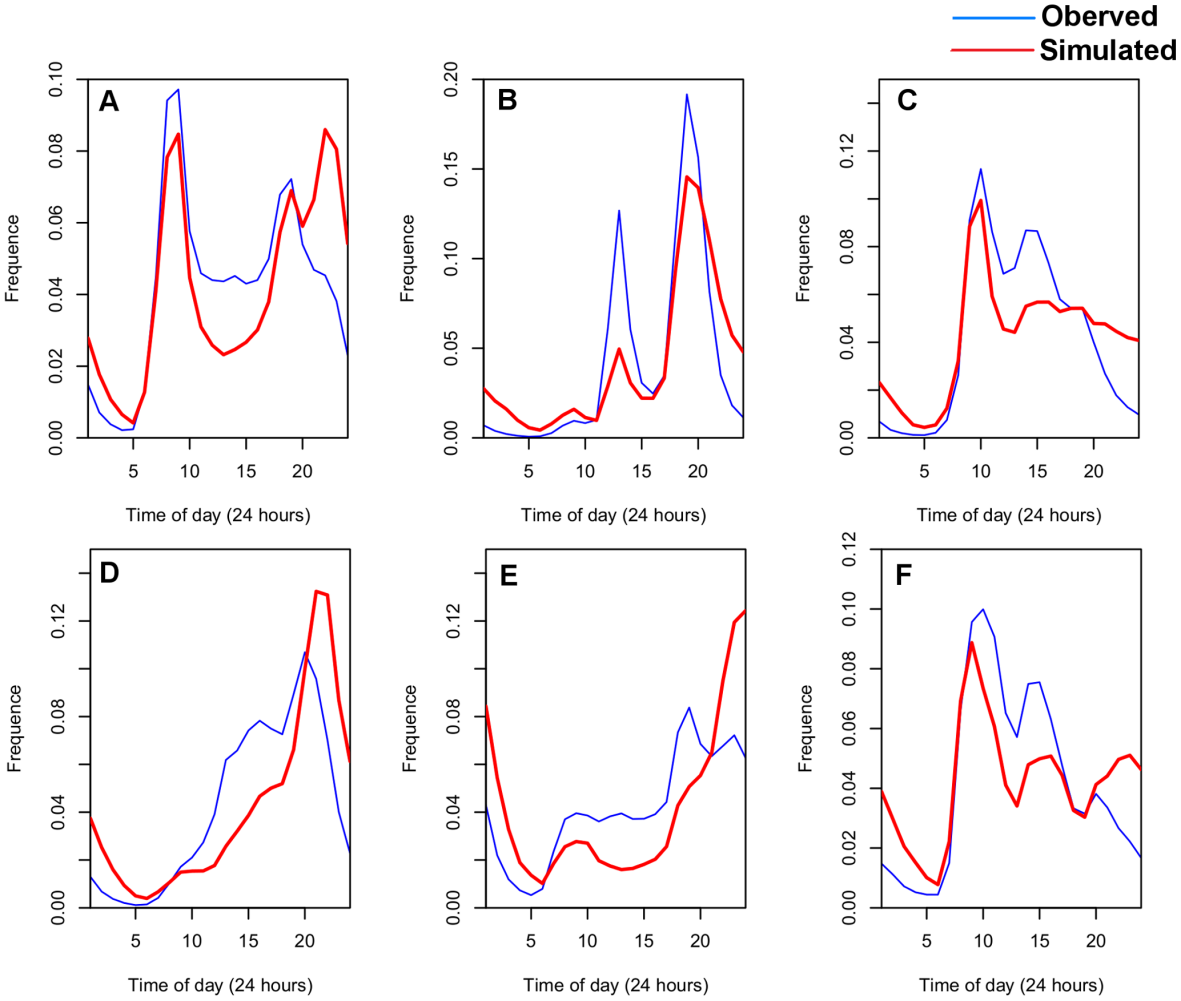
Comparison between temporal distributions of observed and simulated categories. a) Transportation, the Hellinger coefficient is 0.976. b) Dining, the Hellinger coefficient is 0.950. c) Work, the Hellinger coefficient is 0.969. d) Entertainment, the Hellinger coefficient is 0.956. e) Home, the Hellinger coefficient is 0.960. f) Other, the Hellinger coefficient is 0.973. Although deviations still exist in the simulated ones, the deviation values are only a few percent. Besides, all simulated results have similar peak shapes to the observed ones.

As shown in the Figure 8, the Hellinger coefficients for distance distributions are 0.8829, and a peak also exists between 30 km and 40 km indicating that the proposed model interprets the observed distance distributions well. However, the distance distribution cannot ensure the location of activity is correct, therefore, the spatial cluster is brought in to examine this issue. As Figure 9 shows, the spatial distribution of the simulated successor activities is largely similar to the observed one when the Hellinger coefficient is 0.8430. However, it does not fit well in some areas. We conjecture that the reason is the individuals will choose some activities according to their own preferences, which will not be influenced by the geographical impacts or the distance decay effects. Besides, Figure 10 illustrates that the simulated data's trend line matches well with the observed one, and the Hellinger coefficient is 0.9803. Although the simulated data's trend line well matches the observed one, a deviation (about one hour) still exists between two peaks. We conjecture the reason is that the proposed model uses a one-hour time interval. In evening, the check-in activities are more frequent so that a person may check in several times at different places during one hour. According to the proposed model, however, the successor activities are assumed to occur in the next hour, leading to a delayed peak. Lastly, to verify the travel demands intensity distribution in time dimension, we compare the simulated results with the observed ones (Figure 11). All of the simulated curves have high Hellinger coefficients (>0.95) comparing with the actual ones, indicating the proposed model can simulated the travel demands intensity in time dimension well.

## Discussion

Current human mobility studies have paid less attention to activities, due to the lack of explicit large scale activity information data. Fortunately, as social media services have become increasingly used in the past few years, they have also become an indispensable part of many people's lives to record life footprints, including both locations and travel demands. Therefore, social media check-in records have provided a unique opportunity to combine activity-based analysis with movement-based approach in order to study intra-urban human mobility patterns on a large scale. In this study, we utilized these two approaches in combination to reproduce the intra-urban human mobility patterns using the social media check-in data collected from Shanghai, China. By the mechanism of agent-based modeling, the results show that the simulated patterns fit the actual distribution of observed movements well. Hence, our model has illustrated the following three aspects. First, the transition probability between two activities could be regarded as two parts, the transition probability between TTDs and the movement between locations. Second, the travel demand varies over time and is affected by the predecessor activity purpose and the predecessor time. Last, the travel demands could be divided into two categories: LMA and LSA, according to whether the demand is associated with fixed location or not. When one person chooses his/her next stop, the LSA would be affected by not only the distance decay but also the geographical impacts. On the contrary, the LMA has no need to consider the transition probability between locations. As a consequence, there are three trip patterns, judged by the combination of predecessor activity type and the successor activity motivation.

Some limitations still exist in this study. The first is the discontinuous characteristic check-in sequence of an individual. Since the life footprints are only recorded when the individual chooses to upload data, we can only obtain a subset of all the activities of an individual during a day. To overcome this, we introduce a mechanism to judge whether two consecutive check-ins recorded by an individual constitute an activity sequence or not. The second issue is the time uncertainty of check-ins, because the time information of one check-in event cannot indicate the exact time when the user arrives at the venue. In order to avoid this shortcoming, we explore the temporal transition relationship between two types of demands rather than simply considering the time of check-ins as the start time, the duration time or the end time. We assume that the time information of one check-in will have a significant impact on the attributes of a successive check-in. Last, we should be aware of the representativeness of check-in data, that is, the check-in users are not well-designed samples of the population. Young people are more likely to post check-in records on social media, suggesting that check-ins do not have the capability to reflect accurate mobility patterns for all age groups.

Although these limitations will confine the representativeness of check-ins records on human mobility research, the check-in data has illustrated the potential abilities to bridge the gap between activity and mobility patterns analysis, and to create models that incorporate both types of analysis to predict human mobility patterns.

There is some literature on mobility patterns at city level based on activity data (e.g. [20], [35]). However, most such studies suffer from a lack of support from empirical movement data and do not pay much attention to the nature of activities. In the proposed model, human activities are divided into LMAs and LSAs, which play different roles in shaping human mobility patterns. The model is well validated by a check-in data set. Compared to existing studies, this research opens up a new avenue for combining the movement-based approach with the activity-based approach using check-ins, and enriches the theory of activity-based models to travel demand analysis with a quantification of transition matrix of activity. More importantly, this approach may positively impact practical systems and applications in urban planning, traffic management, and mobile location-based services from the perspective of activities.
